# Digesting Digestion: An Educational Laboratory to Teach Students about Enzymes and the Gastrointestinal Tract

**DOI:** 10.1021/acs.jchemed.2c00992

**Published:** 2023-01-19

**Authors:** Stephanie Mack, Sarah L. Barron, Alexander J. Boys

**Affiliations:** † Cancer Research UK Cambridge Institute, University of Cambridge, Robinson Way, Cambridge CB2 0RE, United Kingdom; ‡ Department of Chemical Engineering and Biotechnology, University of Cambridge, Philippa Fawcett Drive, Cambridge, CB3 0AS, United Kingdom

**Keywords:** Digestion, Anatomy, Enzymes, Inquiry-Based Learning, Experiential Learning

## Abstract

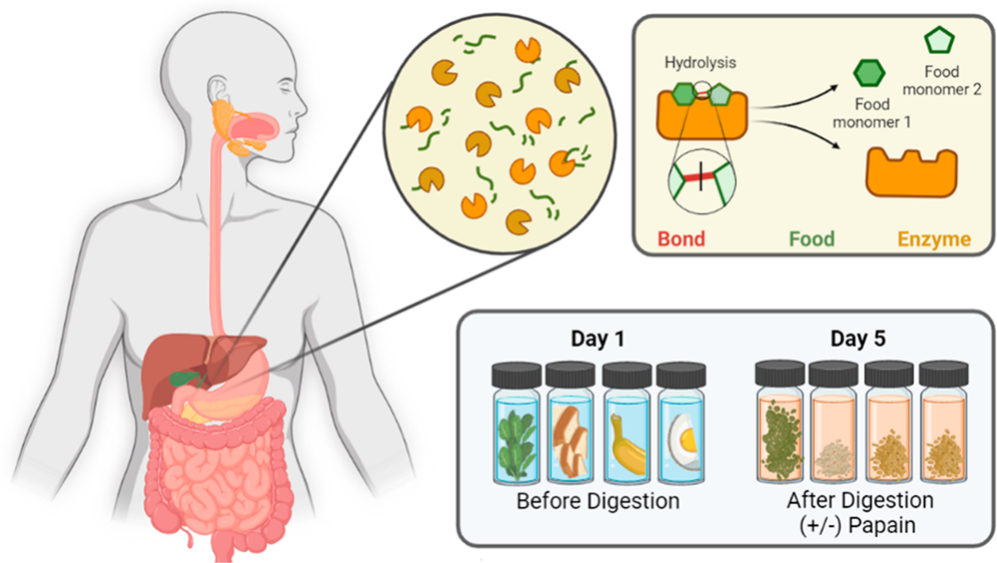

Digestion is a fundamentally important process for an individual’s life. However, the physical process of digestion is hidden inside the body, making it challenging to understand and a particularly difficult topic for students to learn in the classroom. Traditional approaches to teaching body processes include a mixture of textbook teaching and visual learning. However, digestion is not particularly visual. This activity is designed to engage students using a combination of visual, inquiry-based, and experiential learning approaches and introduces the scientific method to students in secondary school. The laboratory simulates digestion, creating a “stomach” inside of a clear vial. Students fill the vials with a protease solution and visually observe the digestion of food. By making predictions about the types of biomolecules that will be digested, students begin to learn and understand basic biochemistry in a relatable context, while simultaneously understanding anatomical and physiological concepts. We trialled this activity at two schools, where we received positive feedback from teachers and students, indicating that the practical enhanced student understanding of the digestion process. We see this lab as a valuable learning activity that can be extended broadly across multiple classrooms around the world.

Digestion of food is simultaneously one of the most easily observable and least visualizable processes in the human body. From an early age, children understand that food is ingested and excreted. However, the process of enzymatic nutrient break down is entirely unobservable. Young students frequently hold misconceptions about the digestive process. Some students believe that all of the food stays inside the body forever, others that food is broken down into smaller pieces mechanically but not chemically, and that food is turned directly into blood.^1−4^ Frequently children believe that only the mouth and stomach are involved in the digestive process and do not link the process to excretion.^1−4^ While students do not necessarily connect enzymatic breakdown of nutrients to the digestive process, they are aware that they need nutrients and that they must eat a variety of foods to get these different nutrients.^5,6^ Understanding digestion requires integrated knowledge of biochemistry and anatomy, subjects infrequently taught together.^7^ There is need for innovative classroom activities that synthesize these subjects in order for students to grasp the entirety of the digestive process.

Standard teaching methods for biochemistry and anatomy utilize teacher-led lectures. In this style, students frequently memorize information but do not gain an understanding of the material.^8^ An entirely active learning curriculum can facilitate learning over passive lectures; however, this can be difficult to implement outside of a university.^9^ Visual learning has proven to be an effective teaching technique which allows students to fully engage with the subject at hand, enhance communication skills, and facilitate the communication of complex topics.^10,11^ Inquiry-based learning is frequently used in the classroom to help students understand scientific concepts,^12−20^ where students form their own hypotheses and use the scientific method. Experiential learning utilizes the pillars of concrete experiences, reflective observation, abstract conceptualization, and active experimentation,^21−23^ to cement new concepts for students. These teaching practices individually promote curiosity and immersion into complex subjects. Combining visual, inquiry-based, and experiential learning is challenging as it requires considerable effort on the part of the instructor, which has limited its usage in the classroom.^24,25^ However, short, predesigned teaching laboratories significantly decrease the barrier to implementation on behalf of the teacher, allowing them to focus on students’ engagement in the classroom. This digestion laboratory integrates visual, inquiry-based, and experiential learning as a tool to synthesize biochemistry and anatomy concepts in a secondary school classroom.

We have developed and implemented an experiential learning laboratory that will improve students understanding of digestion. The laboratory and corresponding teaching module will facilitate a student’s ability to synthesize anatomy and biochemistry concepts. The laboratory includes a detailed teaching module, activity sheet, student handout, question and answer sheet, and final evaluation that teachers can use to aid implementation and assessment. Over three class periods, students are exposed to a visually engaging laboratory to understand the normally opaque process of digestion. Discussions have also been designed to accompany each day, combining traditional teaching methods with a predominantly inquiry-based approach. The students are asked to form their own hypotheses based on their pre-existing knowledge of the macromolecular content of food, observe the digestion of select foods, and compare their observations to their initial hypotheses. At the end of the experience, the students will be exposed to the scientific method and integrate high-level concepts. We demonstrated this laboratory to numerous students at multiple schools to understand its effectiveness as a learning mechanism. We used survey data to tabulate the change in students’ view of their understanding of the digestive process and to receive feedback from teachers. Our laboratory teaches students about digestion in the human body, the structure and function of enzymes, and the process by which enzymes break down food.

## Experimental Overview

Inspired by the grimly fantastical case of Dr. William Beaumont and his patient, Alexis St. Martin,^26^ we have designed a classroom laboratory to offer a window into the digestive system. We have chosen to represent the upper digestive tract on the benchtop, particularly the mouth and stomach. Stomach acid, typically consisting of pepsin and hydrochloric acid, is modeled using an aqueous solution of papain, a protease found in papaya fruits. We have chosen to use papain to model pepsin because both enzymes are endoproteinases that cleave peptide bonds. While pepsin digests a broader range of amino acid sequences than papain, pepsin is activated by hydrochloric acid, and considering the potential hazards of hydrochloric acid use, papain is a relevant substitute. Further, papain is inexpensive, which allowed us to broaden the audience with which this laboratory could be implemented. Papain is commonly available as a dietary supplement and has been used for centuries in South American cuisine as a meat tenderizer. Mechanical digestive mechanisms, such as mastication and peristalsis, are stimulated by the students breaking the foods into smaller pieces prior to placing the foods into the vials and shaking the vials every class period. The students make predictions about how the protease will impact the digestion of these foods at the end of the experiment, and the students compare the breakdown of different foods in water and papain solution and reflect on their initial hypotheses.

This laboratory was designed to introduce digestion, particularly biochemistry and anatomy to secondary school students in an engaging and visual way. We have targeted the laboratory and supporting documents to the level of Year 10/Year 11 students in the United Kingdom (14–16 years old). However, the intended age group of the students can vary depending on how much additional information teachers wish to include coinciding with coursework. Younger students can benefit from a modified version of the laboratory with portions of the practical pre-prepared by the teacher or turned into a demonstration, which maintains the visual learning component.

We examined the Assessment and Qualifications Alliance (AQA) standards, an organization that qualifies the General Certificate of Secondary Education (GCSE) examinations in the United Kingdom to determine for which age group this laboratory would be most appropriate. At this stage students should be familiar with the basic digestive process from past years, where the concepts of enzymatic digestion can be newly explored in depth. Students are exposed to different aspects of the digestive process from Key Stage 2 of the National Curriculum of the United Kingdom. The laboratory is also compatible with Next Generation Science Standards (NGSS), which are standards recognized across the United States for K-12 science education, specifically with the following NGSS standards: HS-LS1-2, HS-LS1-6, HS-LS1-7 (text for these standards can be found in the Supporting Information).

The lab was designed to be accessible to schools of any resource, with minimal practical items necessary for purchase. The authors supplied all of the materials for the demonstrations outlined below, but implementation without author involvement would require the purchase of clear disposable vials, papain powder, and common food items. The price required for the laboratory can be broken down as follows: 10£ (11$) for food (usable for ∼1 class activity), 20£ (22$) for the vials (usable indefinitely), and 20£ (22$) for papain (usable for ∼30 classes).

## Laboratory Design

**Figure 1:**
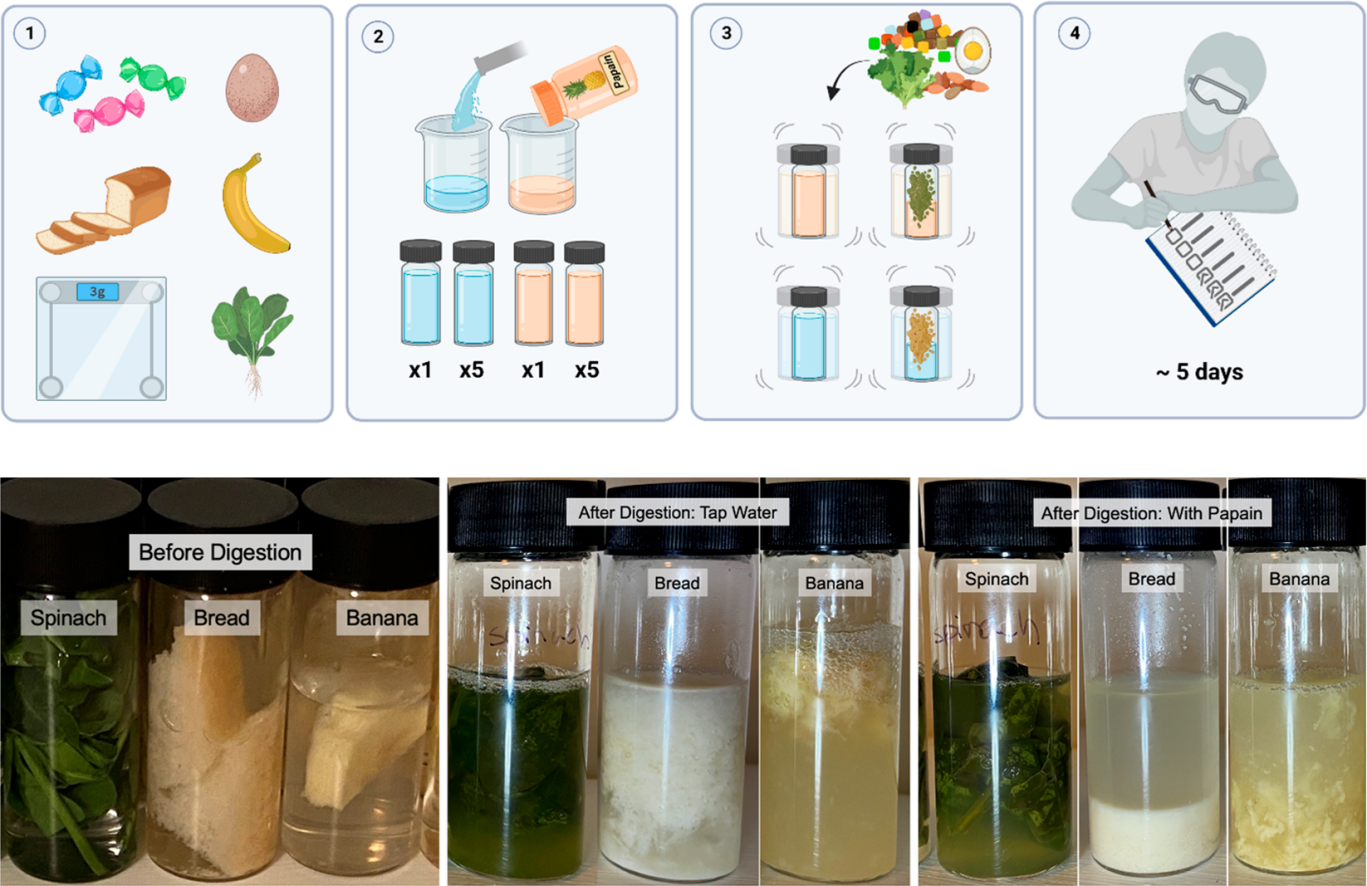
(Top) Summary of digestion lab experimental setup: In the first class period, students are provided with five types of food that are expected to break down at different rates in a solution of papain, the digestive enzyme. Using 12 glass vials, the students prepare a papain group (6 vials) and a tap water group (6 vials). The food is weighed out into 3 g portions and transferred to vials. The two groups allow the students to deconvolute enzymatic breakdown from hydrolysis over the observation period. One vial from each group only contains solution (no food), to illustrate the concept of an experimental control. Vials are shaken, and students record hypotheses and initial observations. (Bottom) Vials before and after digestion. Testing: Over the course of three class periods (5 days), students shake the vials and record observations. The vials are kept at room temperature during this time. Analysis: At the end of the time course, the students compare and contrast degradation between water and papain groups for each food. They are prompted to think about what molecules make up the different types of foods and how that correlates with protease digestion. The students compared the results to their initial hypotheses and answer worksheet questions to reinforce the biological processes occurring in the lab.

The digestion laboratory was designed and tested to observe which foods break down when exposed to papain (Figure 1). Foods with a high protein content are expected to break down rapidly while foods with less protein remain undigested over the course of the laboratory. Each of these foods were chosen because of their different macromolecular compositions. Sweets are composed mainly of carbohydrates and should dissolve readily in water. More complex carbohydrates like bread are digested by amylase enzymes in saliva. However, the structural component of bread, gluten, is a protein. Thus, papain will have a pronounced effect on bread digestion but will not release nutrients. The decomposition of bread in papain is point of reference for students as they may be familiar with gluten-free products. The cell walls in unprocessed plants like spinach and bananas provide some protection from digestive enzymes, and fiber is mostly broken down by bacteria in the small intestines. Eggs are high in protein content and are readily broken down by the body by the major digestive proteases: pepsin, trypsin, and chymotrypsin. As such, these food groups provide a spectrum of biomolecular content through which the students can visualize and understand the digestive process.

The students were asked to set up an experiment where they placed the different types of food into clear glass vials with a solution of papain or water. On Day 1, the students set up the experiment, made predictions about decomposition based on their preexisting knowledge of macromolecules present in the foods, and were taught the history of digestive medicine. Day 2 has the students examine the vials and record observations. They were then taught anatomy and biochemistry for the digestive system. The final day was the experimental wrap up where students made final observations and a class discussion was led surrounding their findings. They were taught about nutrient absorption in the gut, the microbiome, and diseases relating to misfunction of the gastrointestinal system.

At the end of the activity, most foods have undergone a visual amount of digestion. The sweets and bread are typically entirely digested into small particles. The banana and the egg partially dissolve in the papain group. The spinach remains the most intact of all the tested foods in both categories. These differences are recorded by the students and linked back to their initial hypotheses. The actual results will vary depending on how the students aliquoted the foods. Some groups included the crust of the bread, a denser and thus harder to break down portion. Others decided to portion their foods into small pieces which mimics chewing and increases the exposed surface area, thus increasing digestion rates. We chose to allow students some degree of choice in their setup of the laboratory, which we found to assist in the overall discussion at the end of the activity, as students were able to observe and discuss experimental variability. Other foods can be substituted to customize the laboratory for a particular classroom and to avoid any food allergies.

We developed documents to assist teachers’ implementation of this laboratory in their classrooms. We wrote a teaching module explaining the activity that includes an accompanying lesson outline. Teachers can refer to the module for key concepts on digestion and anatomy and when to incorporate these into their lesson plans. In brief, digestion starts in the mouth with mastication and amylase action, and then moves to the stomach for both acidic and enzymatic breakdown. Finally, the food moves through the intestines where food is further digested by enzymes, trypsin and chymotrypsin. Nutrients are then absorbed into the body before excretion. We have also included a laboratory worksheet so students could independently set up the experiment, handouts so students can actively engage in the lesson on digestion and enzymes, and a summary work sheet that teachers can use as a final assessment. The handouts consist of the chemical structures of major macromolecules (proteins, fats, carbohydrates, and nucleic acids), a schematic of the digestive tract, and information on the microbiome and diseases of the intestines (sheets are included in Supporting Information).

## Laboratory Testing

The authors first tested the construction of the activity, piloting additional conditions and foods including apples and kale. White vinegar was initially used in an attempt to mimic the acidic environment of the stomach. However, the addition of acid inactivated the papain, resulting in markedly reduced digestion. The activity was purposed into a live demonstration and debuted at the Cambridge Science Festival to an online audience. The video from the Cambridge Science Festival has been uploaded to YouTube and currently has had over 300 views. Following the successful execution of this laboratory in a remote-learning scenario, the authors implemented the teaching laboratory in two secondary schools in the UK that vary in demographics and resources: an academy in an urban city with 31% of students eligible for free meals (School 1) and a free school in a rural town with 15% of students eligible for free meals (School 2) [households with no more than £16,000 in capital are eligible for free meals]. Both schools were rated by the Office of Standards in Education (Ofsted) with School 1 earning a rating of “Good” and School 2 of “Outstanding.” At School 1 the students were in years 11–13 (aged ∼15–18, *n* = 14), and at School 2 the students were year 10 (aged ∼13–14, *n* = 26). The authors brought all materials to implement the lab and took over teaching responsibilities for the course of the laboratory. The laboratory was performed by a total of *n* = 40 students (*n* = 14 students at School 1, *n* = 26 students at School 2).

The lessons were tailored by the authors to the individual schools as the ages of the students and the lesson lengths were different. School 2 had a larger and younger population of students than School 1 which required the authors to make minor adjustments to the laboratory. School 2 students required more time to accomplish the practical aspects of the activity, and the Lab Activity Sheet was outlined in detail at the start of the class period. Before the laboratory lessons began, the authors asked the students to fill out a survey that consisted of three questions with a Likert response scheme.^27^ At the completion of the experiment, the students were given a second survey with the same three questions plus one additional question. The surveys were intentionally designed to be short with close-ended questions to ensure a high response rate from student populations.^28^ These surveys were used to assess the students’ perceived knowledge of biochemistry, digestion, and anatomy before and after the laboratory to better understand how much students learned over the laboratory and in which subjects the activity imparted the most knowledge. The students were asked to respond to the following statements:

(1) I know how my gastrointestinal tract is arranged
(2) I know how digestion works in the body
(3) I know what enzymes are and how they work
(4) I enjoyed this laboratory

## Data Analysis

Responses from the student surveys were recorded and analyzed in GraphPad Prism version 8.4.3. Ambiguous or incomplete responses were not included in the results (*n* = 2 from School 1 and *n* = 6 from School 2). A two-tailed unpaired *t* test was used to perform statistical analysis when comparing before vs after (95% confidence interval).

## Hazards

General safe laboratory practices should be observed, as in any laboratory setting. Students should not consume any laboratory items. While papain is edible and used in cooking, papain powder may cause skin and eye irritation and any exposure should be thoroughly washed. The teachers can mitigate these risks by making up a stock solution of papain at the concentration indicated and having the students only work with the solution. The authors prepared a stock papain solution when implementing this laboratory. Food allergies should be seriously considered before beginning the lab. As the students are not eating the foods provided, particular attention should be given to students with severe allergies as some particulates may become airborne when aliquoting the foods into vials. Allergy inducing foods can be replaced with other food of similar macromolecular composition.

## Results

**Figure 2:**
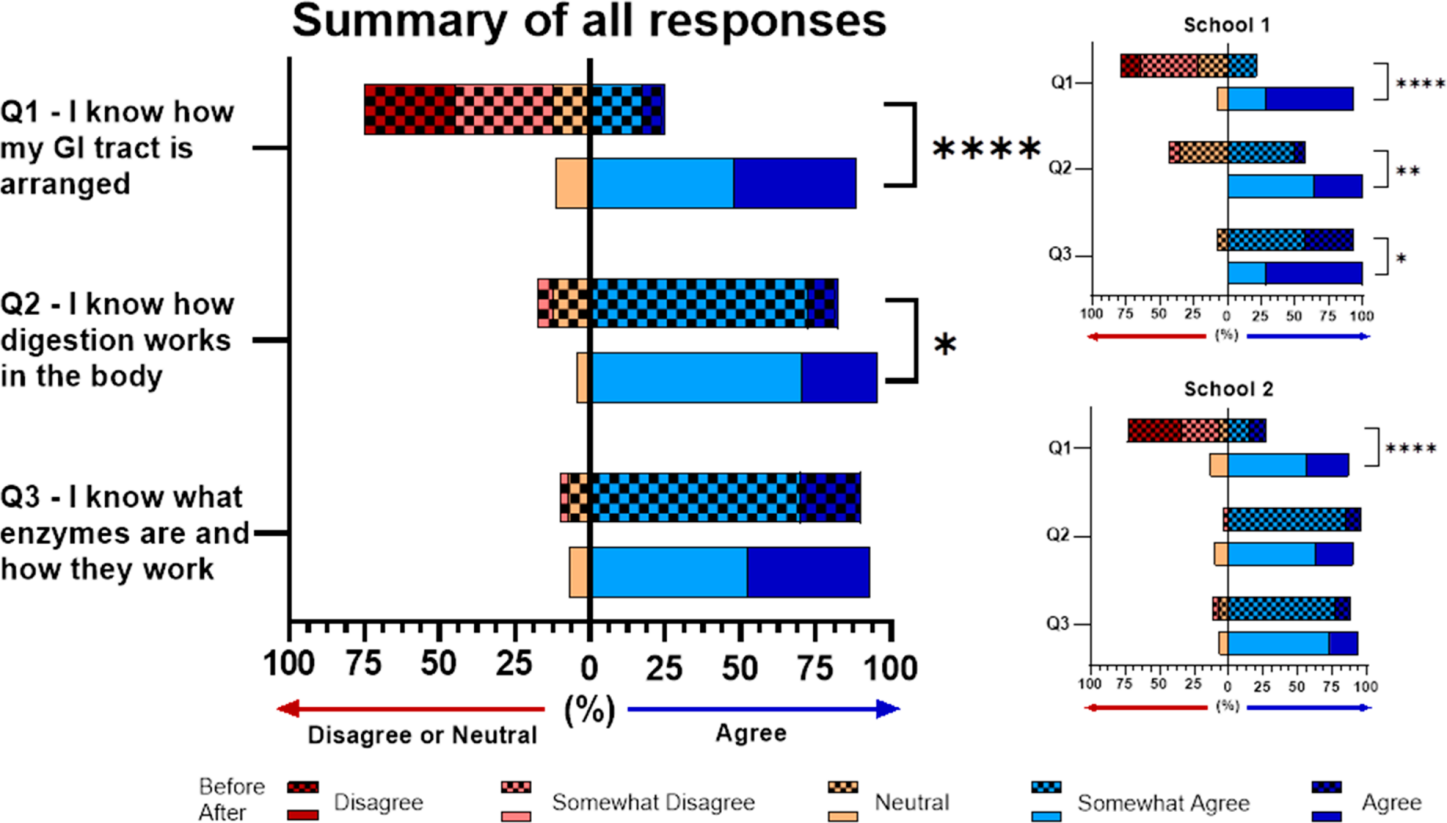
Overall students significantly improved their understanding of GI tract anatomy (*p* < 0.001; ****) and how digestion works in the body (*p* = 0.0121; *). School 1 significantly improved their knowledge on how digestion (*p* = 0.0032; **) and enzymes (*p* = 0.0474; *) work in the body. Statistical analysis was performed using an unpaired *t* test for each question where *n* = 14 for School 1 and *n* = 26 for School 2.

**Figure 3:**
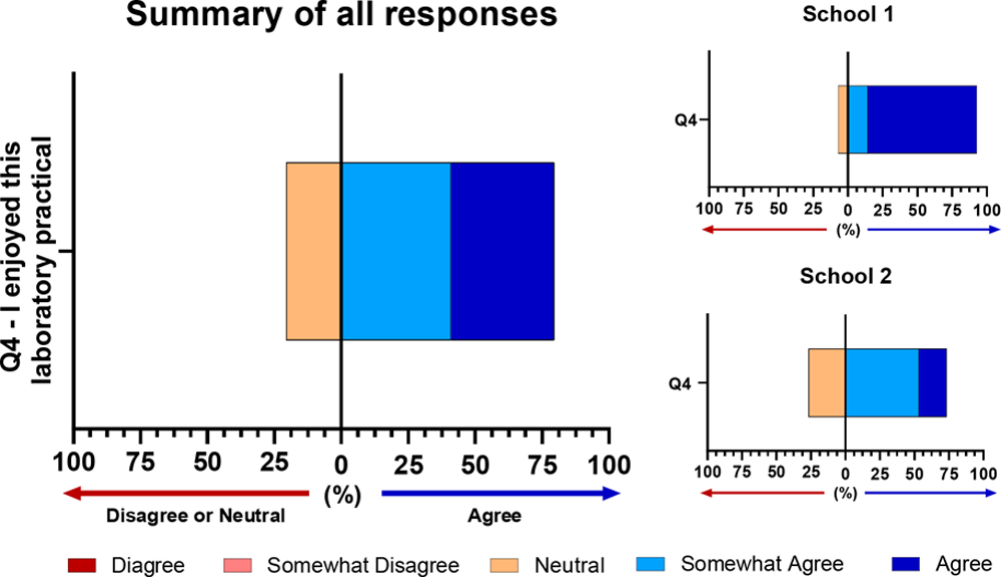
Overall students enjoyed the lab practical with 79.5% of students reporting a positive experience. 92.86% of students from School 1 and 73.33% of students from School 2 reported a positive experience. *n* = 14 for School 1 and *n* = 26 for School 2.

The students’ responses were quantified to determine the success of the laboratory using a Likert scale (1, Disagree; 2, Somewhat Disagree; 3, Neutral; 4, Somewhat Agree; 5, Agree). A response was considered to be positive when the response was either “Somewhat Agree” or “Agree” and considered negative when the response was “Neutral”, “Somewhat Disagree”, or “Disagree”. Across all learning objectives assessed through the survey (anatomy, digestion, enzymes), the students reported an increase in understanding. Anatomy was the subject that students had the least prior knowledge about, with only 25% believing they knew how their gastrointestinal tract was arranged (Figure 2). Enzymes and digestion are two subjects that students learn about throughout their education, starting from Key Stage 2 in the National Curriculum of the UK, and accordingly, 82.5% of all students agreed with the statement that they know how digestion works in the body and 90% of students stated that they knew what enzymes were and how they work (Figure 2). At the end of the activity, knowledge of anatomy significantly increased to 88.6% (*p* < 0.001). The dramatic increase was seen in both schools (21.4% to 92.9% in School 1 and 26.8% to 86.7% in School 2) (Figure 2). Students at School 1 stated they were less knowledgeable about how digestion works in the body before the activity (57.1%), and this significantly increased to 100% of students reporting a success on their postlab survey (*p* = 0.032) (Figure 2). School 1 also significantly improved their knowledge of enzymes, from 92.8% to 100% (*p* = 0.047) (Figure 2). The changes in responses for digestion and enzymes were not significant for School 2. Overall, the students liked the laboratory experience with 79.5% of all students saying they enjoyed the activity (Figure 3).

## Discussion

We have developed a laboratory to facilitate learning the digestive system in secondary school-aged students. Many young students harbor fundamental misunderstandings about digestion and do not link enzymatic action to the digestive process.^1−4,6^ To gain a full understanding of digestion, students need to know the anatomy of the gastrointestinal tract and essentiality of enzymes to digestion. The laboratory and accompanying teaching module allow students to visualize the digestive process by creating benchtop “stomachs” to observe enzymatic action. The combination of visual, inquiry-based, and experiential learning provides the students with a unique learning experience and increases their knowledge of a complex topic. The laboratory was paired with lectures on biochemistry, physiology, and anatomy which reinforces concepts learned over the course of the experiment and allows students to benefit from multiple different modes of teaching.

Anatomy was the most improved subject, and a significant increase was seen in both schools. This is likely because anatomical lessons are typically not carried out at the secondary school level and are reserved for undergraduate studies. Even though students came into the activity with self-reported foundational knowledge in digestion and enzymes, participation in the laboratory continued to increase their understanding of these complex topics. School 1 enjoyed the activity more so than School 2 (92.9% and 73.3%, respectively). This could be attributed to the smaller class size in School 1 which facilitated more interaction between the instructors and the students. Further, the smaller class size encouraged students to participate in classroom discussions.

The instructor at School 2 asked their students for written feedback at the end of the laboratory. Many students thought the practical was engaging, fun, and interesting. One student wrote “it was fun and a great learning experience for me and my peers,” and another said, “the activities were engaging and fun.” Others “enjoyed how different it was to normal lessons.” The lesson left an impact with one student saying “It was fun but also informative. Although the food in papain and water are disgusting but still I learnt lots of new stuff.” Finally, the students noted that they learned a considerable amount of information in a short time and enjoyed the challenge, stating, “learnt a lot about the digestive system in only a short amount of time,” and “we can get to understand how our digestive system works.”

The teachers we worked with were similarly excited by the learning experience. At School 1, the instructor was surveyed to inquire about the effectiveness of the laboratory as a teaching mechanism in their classroom. The teacher thought their students found the laboratory engaging and that it was an effective mechanism for teaching digestion. They also included the comment that “this lab has already been added to our SOW [scheme of work] so thank you for sharing with us.” The instructor also thought the teaching resources provided with the activity were useful saying “good choice on resources—I particularly like the chem sheet to show arrangement of atoms and the history element.”

This study is limited by a few factors. The activity was trialed in two secondary schools and would be improved by feedback from more students and teachers. A full understanding of the effectiveness of this laboratory as a teaching tool is restricted to the queries set out in the survey. As we had limited timeframes to work at these schools, we could only use short surveys that could be implemented at the beginning/end of a class period. The surveys would have been improved with further questions to gain a better assessment of student knowledge prior to performing the laboratory. While the question discussing anatomy seems to have been somewhat accurately answered, given that this subject is not covered in typical curricula, the students had not, according to their teachers, performed considerable coursework on enzymatic action or digestion, which is not reflected in the preassessment survey. Other areas for improvement of the laboratory focus on customization that can be easily implemented by teachers in different classrooms. For example, the laboratory could use foods that may be of direct interest to the students, such as more processed foods or school lunches. Heat could be included as an additional element as the human body functions at higher than room temperature. The papain source used in this activity did not have specific units of active enzyme (how much enzyme is used to convert 1 μmol of substrate in 1 min under optimal conditions). Enzyme concentration, either marked by weight or by activity can be varied and discussed. Further, as other digestive enzymes like amylase are active at different locations along the GI tract, these enzymes could be incorporated as separate experimental groups to visualize how each enzyme can interact with certain macronutrients in classrooms that have more intensive laboratories and available resources.

The laboratory materials include a traditional written assessment for teachers to evaluate students’ understanding of the digestive system at the conclusion of the activity (Supporting Information). The authors used the final assignment as a worksheet that the students could use to self-assess their learning over the course of the laboratory instead of having the teachers incorporate the outcome into their grades. This approach fostered engaging questions and discussion from the students while the answers were reviewed with the entire class.

The laboratory was designed to be flexible and accessible. The authors aimed the activity and teaching resources to secondary school children. However, this activity can be altered at a teacher’s discretion. For example, at the primary school level where students are less able to set up an experiment independently, teachers can do the set up while students are still able to formulate hypotheses, make observations, and draw conclusions from their observations. This allows the lab to be accessible to students at different levels of education. Our choice of materials was intentional to facilitate schools of lower resources to be able to run the activity and have their students benefit from the unique learning experience, enabling implementation of this lab for schools with students of different demographics and backgrounds.

## Conclusion

Students across the globe have similar and fundamental misunderstandings of digestion. Generation of laboratories such as this one can help provide a comprehensive understanding of digestive processes and ideally facilitate the general population’s understanding of nutrition.^1−6,29^ We have designed and implemented a unique laboratory that utilizes visual, inquiry-based, and experiential learning tools to improve secondary school children’s understanding of the digestive system. Including traditional teacher-led lessons into a hands-on laboratory facilitates deep understanding of complex topics. We saw that the laboratory was an overall success as students demonstrated increased knowledge in anatomy, biochemistry, and digestion. Students said they enjoyed the experience saying, “it was fun but also informative”. The provided teaching materials make this activity easy to implement in any secondary school classroom, decreasing the burden on teachers that comes with including visual, experiential, inquiry-based activities into their lesson plans. The activity can be simplified or expanded depending on the resources of the school and the age of the students. The accessibility of the laboratory, demonstrated by the immediate inclusion of this activity in School 1’s curriculum, suggests its suitability for wider incorporation in other classrooms. Through the use of this laboratory, we aim to facilitate students’ understanding of complex scientific concepts that are difficult to visualize and promote scientific knowledge and learning for the next generations.

## Supporting Information Available

Final evaluation (PDF, DOCX)
Handout (PDF, DOCX)
Lab activity sheet (PDF, DOCX)
Laboratory module (PDF, DOCX)
Question and answer sheet (PDF, DOCX)

## References

[ref1] Teixeira F. M. What Happens to the Food We Eat? Children’s Conceptions of the Structure and Function of the Digestive System. Int. J. Sci. Educ 2000, 22 (5), 507–520. 10.1080/095006900289750.

[ref2] Rowlands M. What Do Children Think Happens to the Food They Eat?. J. Biol. Educ 2004, 38 (4), 167–171. 10.1080/00219266.2004.9655936.

[ref3] Cakici Y. Exploring Turkish Upper Primary Level Pupils’ Understanding of Digestion. Int. J. Sci. Educ 2005, 27 (1), 79–100. 10.1080/0950069032000052036.

[ref4] Garcia-Barros S.; Martínez-Losada C.; Garrido M. What Do Children Aged Four to Seven Know about the Digestive System and the Respiratory System of the Human Being and of Other Animals?. Int. J. Sci. Educ 2011, 33 (15), 2095–2122. 10.1080/09500693.2010.541528.

[ref5] Gripshover S. J.; Markman E. M. Teaching Young Children a Theory of Nutrition: Conceptual Change and the Potential for Increased Vegetable Consumption. Psychol Sci. 2013, 24 (8), 1541–1553. 10.1177/0956797612474827. 23804961

[ref6] Jahic Pettersson A.; Danielsson K.; Rundgren C.-J. Traveling Nutrients”: How Students Use Metaphorical Language to Describe Digestion and Nutritional Uptake. Int. J. Sci. Educ 2020, 42 (8), 1281–1301. 10.1080/09500693.2020.1756514.

[ref7] Estai M.; Bunt S. Best Teaching Practices in Anatomy Education: A Critical Review. Annals of Anatomy 2016, 208, 151–157. 10.1016/j.aanat.2016.02.010. 26996541

[ref8] Lujan H. L.; DiCarlo S. E. Too Much Teaching, Not Enough Learning: What Is the Solution?. American Journal of Physiology - Advances in Physiology Education 2006, 30 (1), 17–22. 10.1152/advan.00061.2005. 16481604

[ref9] Reich N.; Wang Y. Highly Effective Active Learning in a One-Year Biochemistry Series with Limited Resources. Biochemistry and Molecular Biology Education 2018, 47 (1), 7–15. 10.1002/bmb.21186. 30548908

[ref10] McGrath M. B.; Brown J. R. Visual Learning for Science and Engineering. IEEE Comput. Graph Appl. 2005, 25 (5), 56–63. 10.1109/MCG.2005.117. 16209171

[ref11] Ramadas J. Visual and Spatial Modes in Science Learning. Int. J. Sci. Educ 2009, 31 (3), 301–318. 10.1080/09500690802595763.

[ref12] Cabalsa J. M.; Abraham L. Exploring Biochemical Reactions of Proteins, Carbohydrates, and Lipids through a Milk-Based Demonstration and an Inquiry-Based Worksheet: A Covid-19 Laboratory Experience. J. Chem. Educ. 2020, 97 (9), 2669–2677. 10.1021/acs.jchemed.0c00666.

[ref13] Kim J. Production of Biodiesel from Waste Cooking Oil: A Guided Inquiry Chemistry Laboratory Activity at a Two-Year College. J. Chem. Educ. 2022, 99, 4162 10.1021/acs.jchemed.2c00265.

[ref14] Ghirardi M.; Marchetti F.; Pettinari C.; Regis A.; Roletto E. A Teaching Sequence for Learning the Concept of Chemical Equilibrium in Secondary School Education. J. Chem. Educ. 2014, 91 (1), 59–65. 10.1021/ed3002336.

[ref15] Bethel C. M.; Lieberman R. L. Protein Structure and Function: An Interdisciplinary Multimedia-Based Guided-Inquiry Education Module for the High School Science Classroom. J. Chem. Educ. 2014, 91 (1), 52–55. 10.1021/ed300677t.

[ref16] Pope S. R.; Tolleson T. D.; Williams R. J.; Underhill R. D.; Deal S. T. Working with Enzymes - Where Is Lactose Digested?. J. Chem. Educ. 1998, 75 (6), 761–761. 10.1021/ed075p761.

[ref17] Vroom Redden A. M.; Barton C. M.; Willian K. R. Combined Guided and Open Inquiry Project for an Upper Division Biochemistry Lab: Sugar Content, Enzymatic Properties of Lactase, and the Spoiling Process in Milk. J. Chem. Educ. 2020, 97 (5), 1430–1436. 10.1021/acs.jchemed.9b00926.

[ref18] House C.; Meades G.; Linenberger K. J. Approaching a Conceptual Understanding of Enzyme Kinetics and Inhibition: Development of an Active Learning Inquiry Activity for Prehealth and Nonscience Majors. J. Chem. Educ. 2016, 93 (8), 1397–1400. 10.1021/acs.jchemed.5b00562.

[ref19] Rotjanakunnatam B.; Chayaburakul K. Developing the Conceptual Instructional Design with Inquiry-Based Instruction Model of Secondary Students at the 10th Grade Level on Digestion System and Cellular Degradation Issue. In AIP Conference Proceedings; American Institute of Physics Inc., 2018; Vol. 1923, p 030041. 10.1063/1.5019532.

[ref20] Boys A. J.; Walsh M. C. Introducing Engineering Design and Materials Science at an Earlier Age through Ceramic Cold Casting. J. Chem. Educ. 2019, 96 (1), 104–109. 10.1021/acs.jchemed.8b00404. 31105331 PMC6521834

[ref21] Morris T. H. Experiential Learning–a Systematic Review and Revision of Kolb’s Model. Interactive Learning Environments; Routledge, 2020; pp 1064–1077. 10.1080/10494820.2019.1570279.

[ref22] Weinberg A. E.; Basile C. G.; Albright L. The Effect of an Experiential Learning Program on Middle School Students’ Motivation Toward Mathematics and Science. RMLE Online 2011, 35 (3), 1–12. 10.1080/19404476.2011.11462086.

[ref23] Powell K.; Wells M. The Effectiveness of Three Experiential Teaching Approaches on Student Science Learning in Fifth-Grade Public School Classrooms. J. Environ. Educ 2002, 33 (2), 33–38. 10.1080/00958960209600806.

[ref24] Costenson K.; Lawson A. E. Why Isn’t Inquiry Used in More Classrooms?. Am. Biol. Teach 1986, 48 (3), 150–158. 10.2307/4448241.

[ref25] Boesdorfer S. B.; del Carlo D. I.; Wayson J. Secondary Science Teachers’ Reported Practices and Beliefs on Teaching and Learning from a Large National Sample in the United States. J. Sci. Teacher Educ 2019, 30 (8), 815–837. 10.1080/1046560X.2019.1604055.

[ref26] Markel H. How William Beaumont and Alexis St. Martin Seized the Moment of Scientific Progress. J. Am. Med. Assoc 2009, 302 (7), 804–806. 10.1001/jama.2009.1212. 19690317

[ref27] Joshi A.; Kale S.; Chandel S.; Pal D. Likert Scale: Explored and Explained. Br J. Appl. Sci. Technol. 2015, 7 (4), 396–403. 10.9734/BJAST/2015/14975.

[ref28] Sinkowitz-Cochran R. L. Survey Design: To Ask or Not to Ask? That Is the Question···. Clinical Infectious Diseases 2013, 56 (8), 1159–1164. 10.1093/cid/cit005. 23315318

[ref29] Andersson J.; Löfgren R.; Tibell L. A. E. What’s in the Body? Children’s Annotated Drawings. J. Biol. Educ 2020, 54 (2), 176–190. 10.1080/00219266.2019.1569082.

