# Supplementary material for: Digesting Digestion: An Educational Laboratory to Teach Students about Enzymes and the Gastrointestinal Tract
Source: J Chem Educ. 2023 Jan 19;100(2):907–13. doi: 10.1021/acs.jchemed.2c00992 (PMC9933529; doi:10.1021/acs.jchemed.2c00992)
Supplement: Supplementary file 1 — ed2c00992_si_001.pdf [file ed2c00992_si_001.pdf]

Supporting Information

Final Evaluation

Digesting digestion: An educational laboratory to teach students about enzymes and the gastrointestinal tract

Stephanie Mack[1], Sarah L. Barron[2], Alexander J. Boys[2]*

1. Cancer Research UK Cambridge Institute, University of Cambridge, Robinson Way, Cambridge CB2 0RE, United Kingdom

2. Department of Chemical Engineering and Biotechnology, University of Cambridge, Philippa Fawcett Drive, Cambridge, CB3 0AS, United Kingdom

* Corresponding Author (ab2661@cam.ac.uk)

Enzyme Lab Final Worksheet

1. What role does each biomolecule play in the cell?
    a. Fats
       Long-term energy storage.

    b. Polysaccharides
       Short-term energy storage.

    c. Nucleic Acids
       Information storage

    d. Proteins
       Chemical work / everything else.

2. Match the enzyme to the biomolecule it breaks down:

Lipase (a)	Nucleic acid (b)
Nuclease (b)	Protein (c)
Protease (c)	Fats (a)
Glycoside hydrolase (d)	Polysaccharide (d)

3. What is peristalsis?
   The pulsatile movement of muscles in the GI tract to move food down the tract.

4. Your mouth starts the digestive process. What occurs there?
   The mouth starts the digestive process mechanically by chewing food into smaller pieces. Amylase, also found in the mouth, breaks down starch through chemical digestion.

5. How are proteins digested? What particular enzymes are active in each part of the process and where do they occur?
   Pepsin is activated by acid in the stomach and begins breaking down proteins by cleaving after large amino acids. Trypsin is secreted by the pancreas, is active in the small intestine and cleaves after two positively charged amino acids lysine and arginine. Chymotrypsin is also secreted by the pancreas and active in the small intestine but it cleaves similarly to pepsin, large amino acids.

6. What class of biomolecule is gluten?
   Gluten is a protein.

7. How are trypsin and papain similar and how are they different?
   Papain and trypsin are both proteases. Papain is from the papaya plant and trypsin is generated in the pancreas. The two proteases cleave after different amino acids - trypsin after lysine and arginine papain after leucine and glycine.

8. Explain the process of fermentation and give an example of how it used inside the body and in the food industry.
Fermentation is used inside the gut by bacteria/microorganisms to help break down fibrous/starchy foods to release nutrients; mention of fermentation being used to help preserve/sterilise foods e.g. bread, dairy, alcohol or increasing the nutritional content of food.

9. Label a diagram of the digestive system:

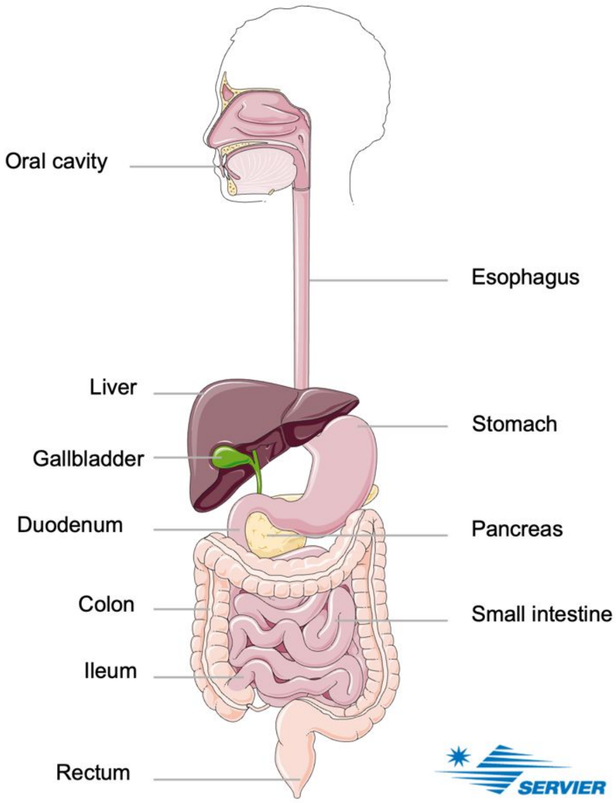

This image is reused under Creative Commons License 3.0

10. Name a disease of the digestive system.
Bonus: Explain how it may lead to malnutrition or related symptoms.
Celiac Disease/Inflammatory Bowel Disease (IBD)/Crohn's Disease, which, if left untreated, lead to malnutrition, weight loss, exhaustion and depression. In Celiac Disease, the immune system is triggered by a protein found within some carbohydrates (gluten) and attacks the lining of the small intestine, leading to flattened and/or damaged microvilli. Without microvilli, very little nutritional content can be absorbed through the intestine and leads to symptoms such as diarrhea, cramps, weakened immune system and in children stunted growth. Luckily, these effects can be reversed by adopting a strict gluten free diet.

11. What part of the GI tract mixes bile, enzymes, and food (chyme) together (be specific!)?
The duodenum (small intestine).

Bonus Questions

12. Trace your favourite meal through the digestive process. Identify where different foods will be broken down along your digestive tract and through what means.
    A hamburger is first broken down in the mouth by mastication. Amylase enzymes will begin the digestion of carbohydrates in the bread. As the mouthful is swallowed, peristalsis continues to mechanically break down the food. When the hamburger hits the stomach, high acidity and pepsin break down proteins. After sufficient time the food mass is moved into the small intestine where trypsin and chymotrypsin continue to break down proteins. Gut bacteria aid in digestion by attacking cell walls from the hamburger's lettuce and tomato. The indigestible waste is packaged in the large intestine and excreted.

13. Papain can be bought in the grocery store. How do you think you could use papain in your cooking?
    Papain is frequently used in South American cooking as a meat tenderiser.
