# Supplementary material for: Digesting Digestion: An Educational Laboratory to Teach Students about Enzymes and the Gastrointestinal Tract
Source: J Chem Educ. 2023 Jan 19;100(2):907–13. doi: 10.1021/acs.jchemed.2c00992 (PMC9933529; doi:10.1021/acs.jchemed.2c00992)

Supporting Information

Handout

Digesting digestion: An educational laboratory to teach students about enzymes and the gastrointestinal tract

Stephanie Mack[1], Sarah L. Barron[2], Alexander J. Boys[2]*

1. Cancer Research UK Cambridge Institute, University of Cambridge, Robinson Way, Cambridge CB2 0RE, United Kingdom

2. Department of Chemical Engineering and Biotechnology, University of Cambridge, Philippa Fawcett Drive, Cambridge, CB3 0AS, United Kingdom

* Corresponding Author (ab2661@cam.ac.uk)

Biomolecule	Structure	Enzyme	Basic Unit
Protein	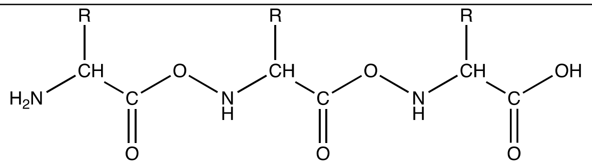	Protease	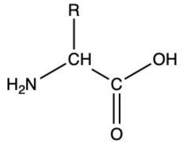
Fat	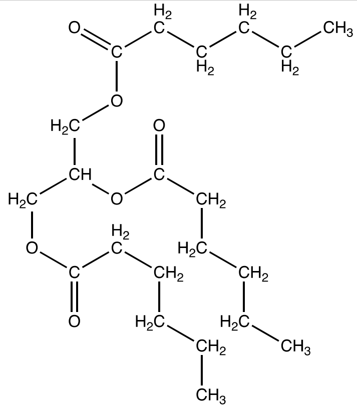	Lipase	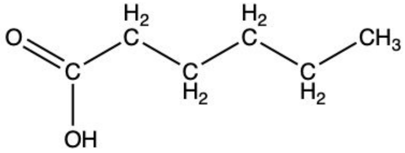
Polysaccharide	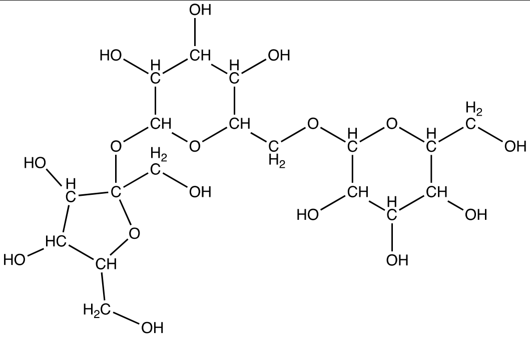	Glycoside Hydrolase	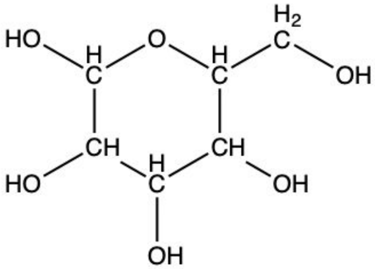
Nucleic Acid	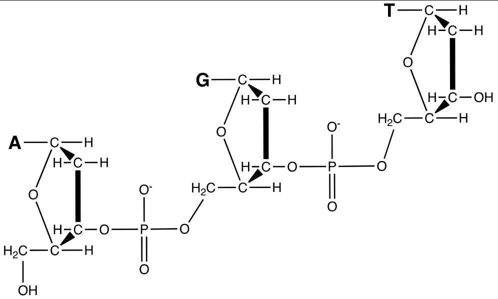	Nuclease	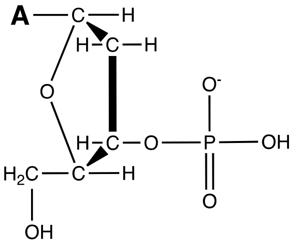

Your Digestive Tract:

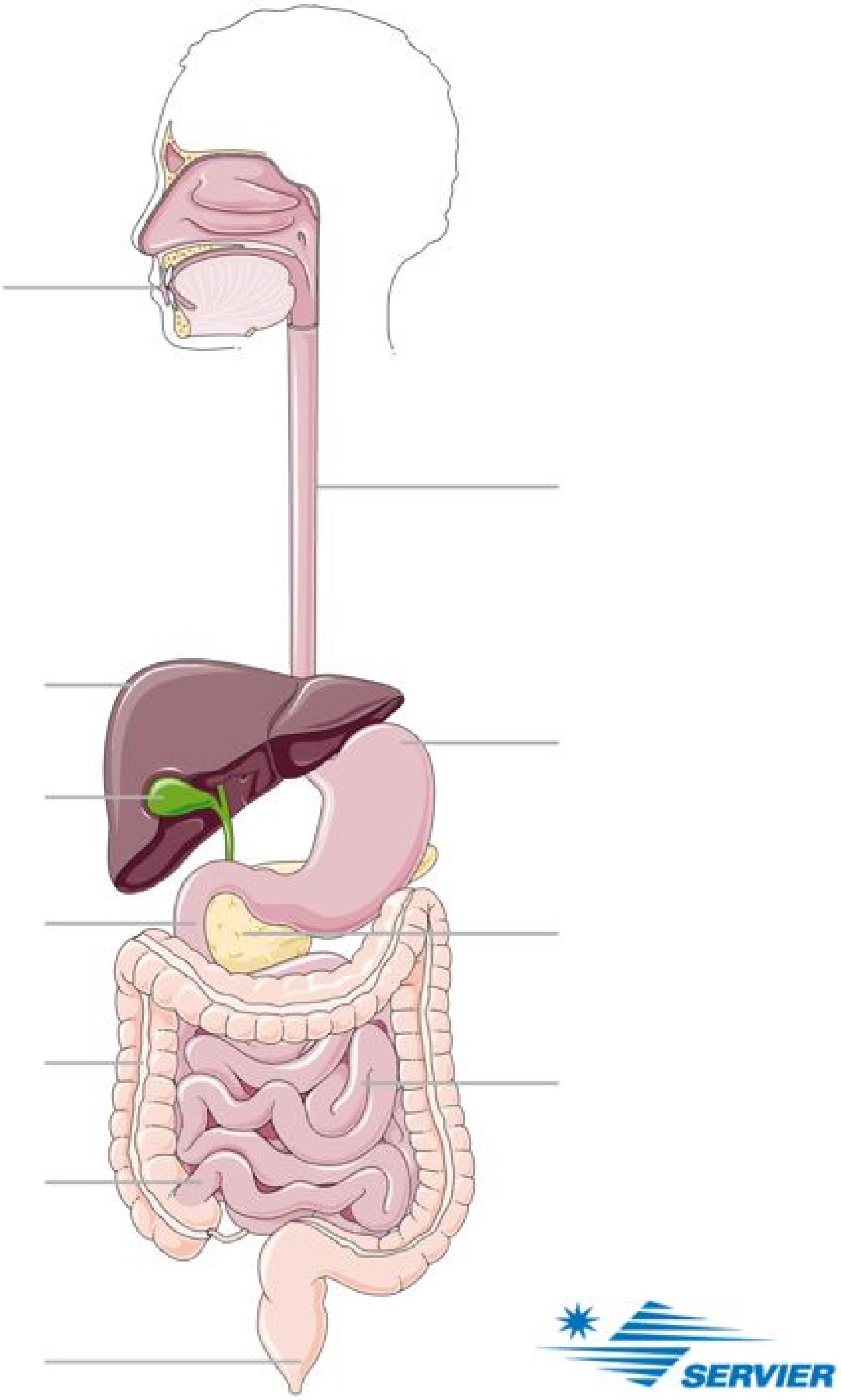

This image is reused under Creative Commons License 3.0

Celiac Disease

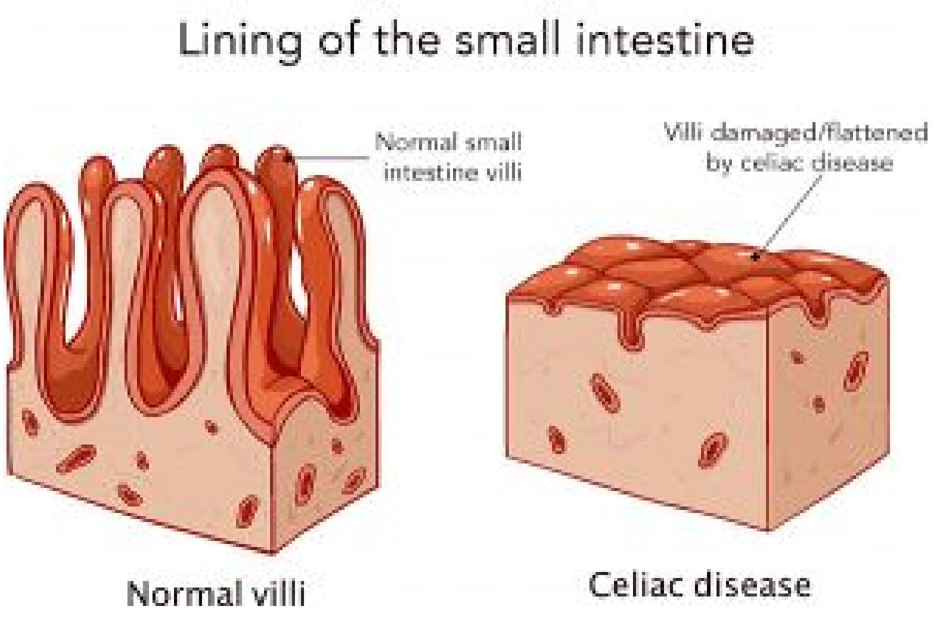

Gut Microbiome

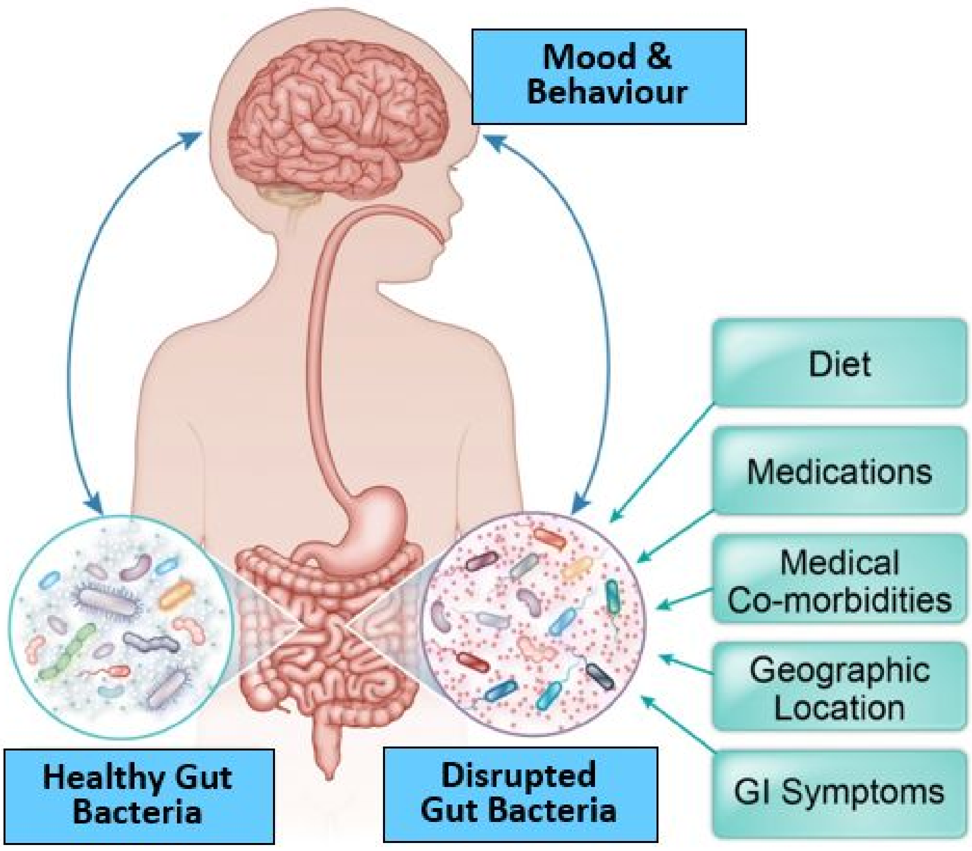

Supplement: Supplementary file 3 — ed2c00992_si_003.pdf [file ed2c00992_si_003.pdf]
