# Supplementary material for: Digesting Digestion: An Educational Laboratory to Teach Students about Enzymes and the Gastrointestinal Tract
Source: J Chem Educ. 2023 Jan 19;100(2):907–13. doi: 10.1021/acs.jchemed.2c00992 (PMC9933529; doi:10.1021/acs.jchemed.2c00992)

**Supporting Information**

Handout

**Digesting digestion: An educational laboratory to teach students about enzymes and the gastrointestinal tract**

Stephanie Mack^1^, Sarah L. Barron^2^, Alexander J. Boys^2^*

1. Cancer Research UK Cambridge Institute, University of Cambridge, Robinson Way, Cambridge CB2 0RE, United Kingdom
2. Department of Chemical Engineering and Biotechnology, University of Cambridge, Philippa Fawcett Drive, Cambridge, CB3 0AS, United Kingdom

* Corresponding Author (ab2661@cam.ac.uk)

| **Biomolecule** | **Structure** | **Enzyme** | **Basic Unit** |
| --- | --- | --- | --- |
| Protein |  | Protease | 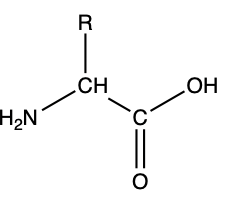 |
| Fat |  | Lipase | 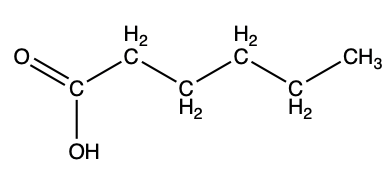 |
| Polysaccharide |  | Glycoside Hydrolase | 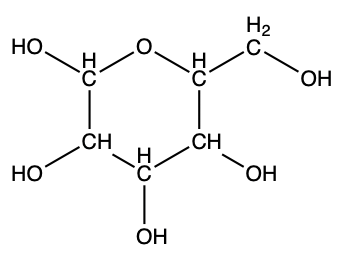 |
| Nucleic Acid |  | Nuclease |  |

**Your Digestive Tract:**


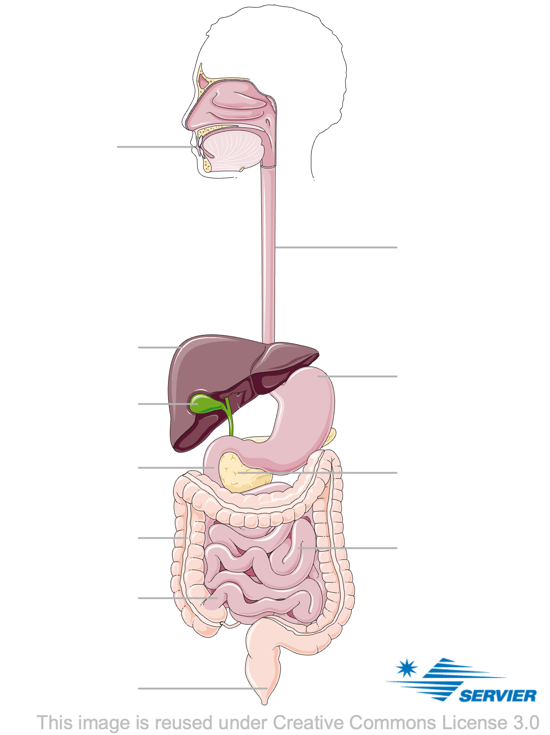


**Celiac Disease**


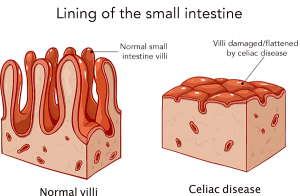


**Gut Microbiome**


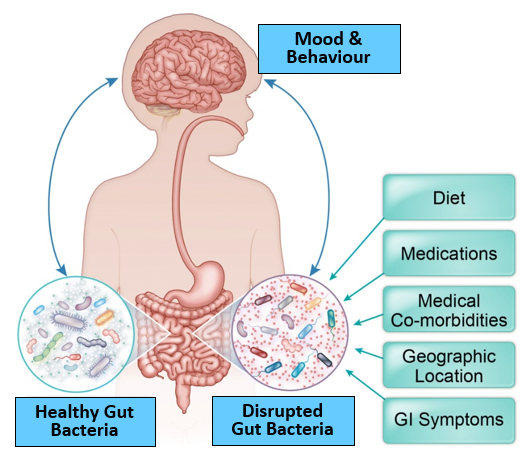

Supplement: Supplementary file 4 — ed2c00992_si_004.docx [file ed2c00992_si_004.docx]
