# Supplementary material for: Digesting Digestion: An Educational Laboratory to Teach Students about Enzymes and the Gastrointestinal Tract
Source: J Chem Educ. 2023 Jan 19;100(2):907–13. doi: 10.1021/acs.jchemed.2c00992 (PMC9933529; doi:10.1021/acs.jchemed.2c00992)
Supplement: Supplementary file 6 — ed2c00992_si_006.docx [file ed2c00992_si_006.docx]

**Supporting Information**

Lab Activity Sheet

**Digesting digestion: An educational laboratory to teach students about enzymes and the gastrointestinal tract**

Stephanie Mack^1^, Sarah L. Barron^2^, Alexander J. Boys^2^*

1. Cancer Research UK Cambridge Institute, University of Cambridge, Robinson Way, Cambridge CB2 0RE, United Kingdom
2. Department of Chemical Engineering and Biotechnology, University of Cambridge, Philippa Fawcett Drive, Cambridge, CB3 0AS, United Kingdom

* Corresponding Author (ab2661@cam.ac.uk)

**How does your body digest food?**

**Background Info:**

Your body’s digestion system consists of a series of tubular organs that serve various functions in breaking down food. Digestion occurs through both mechanical and chemical means. Mechanical motions help break food into smaller pieces and move the processed food through your gastrointestinal tract. Throughout this process, various enzymes act on the chemical constituents of food, breaking them into smaller nutrients and molecules that your body can either use or excrete. This laboratory will focus on the action of enzymes on different food groups so you can see what happens inside your digestive system.

**Objective:**

Observe and record the digestion of various food groups using the enzyme papain. Make predictions of the foods to be most thoroughly digested based on what you have learned.

**Vocabulary:**

- Protein
- Protease
- Papain

**Materials:**

- Sweets
- Bread
- Spinach
- Banana
- Egg
- Papain Powder
- Water
- 12 Glass Vials (with caps)

**Procedure:**

____ 1) Prepare papain solution by mixing 1 g of papain powder into 350 mL of water.

____ 2) Weigh out 3 g of each food in preparation for digestion (2x).

____ 3) Transfer foods into glass vials.

____ 4) Label half of the glass vials (6) for water and half of the glass vials (6) for papain.

____ 5) Transfer 15 mL papain solution into half of the glass vials, with the final vial containing

only papain solution and no food (6).

____ 6) Transfer 15 mL of water into the other half of the glass vials, with the final vial containing

only water and no food (6).

____ 7) Record any initial observations.

____ 8) Shake each vial thoroughly to homogenize the solution.

____ 9) Record further observations.

**Observations:**

**Observations:**

**Observations:**
