# Supplementary material for: Digesting Digestion: An Educational Laboratory to Teach Students about Enzymes and the Gastrointestinal Tract
Source: J Chem Educ. 2023 Jan 19;100(2):907–13. doi: 10.1021/acs.jchemed.2c00992 (PMC9933529; doi:10.1021/acs.jchemed.2c00992)
Supplement: Supplementary file 7 — ed2c00992_si_007.pdf [file ed2c00992_si_007.pdf]

Supporting Information

Laboratory Module

Digesting digestion: An educational laboratory to teach students about enzymes and the gastrointestinal tract

Stephanie Mack[1], Sarah L. Barron[2], Alexander J. Boys[2]*

1. Cancer Research UK Cambridge Institute, University of Cambridge, Robinson Way, Cambridge CB2 0RE, United Kingdom

2. Department of Chemical Engineering and Biotechnology, University of Cambridge, Philippa Fawcett Drive, Cambridge, CB3 0AS, United Kingdom

* Corresponding Author (ab2661@cam.ac.uk)

Digestion

Author(s): Stephanie Mack, Sarah Barron, Alexander Boys

Date Created: 2021

Subject: Biology

Grade Level: Secondary School

Standards:
UK Standards:

AQA General Certificate of Secondary Education (GCSE) (www.aqa.org.uk)

GCSE Biology 8461
(Unit 4.2.2.1 The Human Digestive System)

GCSE Chemistry 8462
(Unit 4.7.3.3 Amino Acids [HT Only])

US Standards:

Next Generation Science Standards (www.nextgenscience.org)

HS-LS1-2 From Molecules to Organisms: Structures and Processes

HS-LS1-6 From Molecules to Organisms: Structures and Processes

HS-LS1-7 From Molecules to Organisms: Structures and Processes

Schedule: 3 – 1 hour classes

**Objectives:**	**Vocabulary:**
Students will learn about digestion in the human body, the function and structure of enzymes, and the process by which enzymes break down food. They will weigh out and produce enzymatic digestion solutions and apply these solutions to a variety of foods representing different food groups. The students will record observations as these foods are digested by the enzymes, while learning about the chemistry behind enzymatic digestion. They will study the association between enzymatic digestion in a laboratory setting versus in the human body.	Enzyme Classes<br>• Lipase<br>• Nuclease<br>• Protease<br>• Glycoside Hydrolase<br>Enzymes<br>• Papain<br>• Trypsin<br>Gastrointestinal Tract<br>• Mouth<br>• Esophagus<br>• Stomach<br>• Duodenum<br>• Jejunum<br>• Ileum<br>• Colon<br>• Rectum<br>Biomolecules<br>Food Chemistries<br>• Fats<br>• Polysaccharides<br>• Nucleic Acids<br>• Proteins<br>Applications<br>• Microbiome<br>• Fermentation<br>• Gluten<br>• Microvilli

**Students Will:**	**Materials:**
Learn the basics of digestion in the human body<br>Review the anatomy of the gastrointestinal tract and associate different organs with different digestive process<br>Study the process of enzymatic digestion, learn about different enzymes and the chemistry by which they work<br>Mix enzymatic digestion solutions<br>Take observations of the digestion of different food groups<br>Learn the chemistry that is occurring in this experiment and make predictions as to which foods will digest most<br>Demonstrate their findings in writing	**For Each Group (2-4 students)**<br>Foods<br>Bread<br>Sweets<br>Spinach<br>Banana<br>Egg<br>Glass Vials (12x)<br><br>**For Class**<br>Papain Powder<br><br>**For Teacher**<br>SDS for Papain<br><br>**Provided by Teacher**<br>Scale (*optional*)<br>Measuring cup<br>Beaker<br>Graduated cylinder<br>Stirrer
**Safety**	Please refer to SDS sheet for papain before using.<br><br>Please check for food allergies among students.

Classroom Procedure Overview:

1st Class:
Introductory content for the class **(Time: 10 mins)**

Overview of laboratory setup **(Time: 10 mins)**

Set up experiment **(Time: 30 mins)**

Record observations **(Time: 10 mins)**

2nd Class:
Record observations **(Time: 5 mins)**

Learn anatomy **(Time: 10 mins)**

Learn food chemistry **(Time: 20 mins)**

Learn chemistry behind enzymatic processing **(Time: 20 mins)**

Applications overview **(Time: 10 mins)**

3rd Class:
Record observations **(Time: 5 mins)**

Lab Wrap-up & Questions **(Time: 10 mins)**

Wrap-up worksheet **(Time: 30 mins)**

Wrap-up worksheet answers **(Time: 10 mins)**

Final questions from students **(Time: 5 mins)**

<u>Science Content for the Teacher per Class</u>:

1st Class:
Background and Historical Context

- Story of Alexis St. Martin and William Beaumont
  - In 1822, St. Martin, a French Canadian man, was accidentally shot in the chest with a musket from 3ft away while visiting a trading post in what is now Michigan, US. The musket shot was so close that St. Martin actually lit on fire, and the shot resulted in the expulsion of many of his internal organs. A local doctor, William Beaumont, was called to examine St. Martin stating that St. Martin would likely not live for any longer than a day or two. However, St. Martin made a miraculous recovery with only one major reminder, a hole in his chest that led directly into his stomach (known as a gastric fistula). Beaumont suggested repairing the hole, but St. Martin, tired of being poked and prodded, asked to be left alone. Over time, a flap healed over the hole, which still allowed access to the stomach. Beaumont took on St. Martin as a kind of living experiment, both supporting and experimenting on him for approximately 10 years. This bizarre event led Beaumont to conduct a series of tests, where he was able to place different foods into St. Martin's stomach, actually observing the digestion of these foods in the stomach as it occurred. Beaumont's (and St. Martin's) work led to some of our earliest understanding of how digestion works, providing details for what stomach acid (or gastric juice) is and does, as well as how it affects different foods.

2nd Class:
Anatomy
Gastrointestinal Tract (GI Tract)

- Series of long, tubular organs that play different roles in breaking down food

- Also called the digestive tract or the alimentary canal (referring specifically to the continuous tubular tract of the gastrointestinal system)
- Major organs of the GI Tract
  - Mouth
  - Esophagus
  - Stomach
  - Small Intestine
    - Duodenum
    - Jejunum
    - Ileum
  - Colon
  - Rectum
- Digestion occurs through two major means
  - Mechanical motion
    - Chewing
    - Peristalsis
  - Enzymatic action

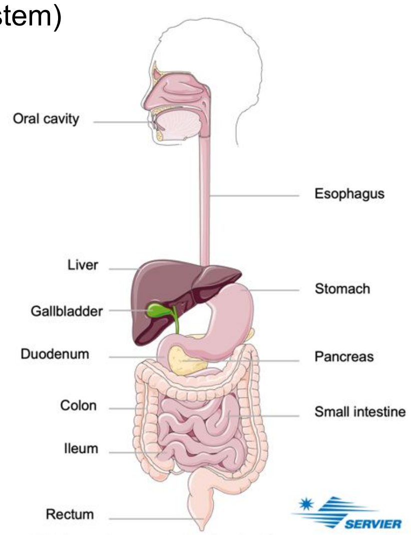

This image is reused under Creative Commons License 3.0

Organ	Mechanics	Function	Notes
Mouth	Chewing	Breaks food into small pieces	Saliva breaks down starches, starting point for digestion
Esophagus	Peristalsis	Move food to stomach	Lower esophageal sphincter acts as valve between stomach and esophagus
Stomach	Mixing	Mixes food with gastric juices, secretes enzymes and acid to break down food, result is called chyme	Upper muscle moves food into stomach, lower muscle mixes food with gastric juice
Duodenum	Peristalsis	Mixes chyme with bile from the gallbladder/liver and enzymes from the pancreas to finish breakdown of food and begin absorption process	Smallest portion of the small intestine (small bowel)
Jejunum	Peristalsis	Absorption of nutrients (sugars, amino acids, fatty acids)	2.5 m in length
Ileum	Peristalsis	Absorbs remaining nutrients (vitamin B12), recycles bile acids	3 m in length
Colon	Peristalsis	Absorbs water, forms stool	Contains large amounts of gut bacteria, which also aid in digestion, vitamin production, and more

Rectum	Muscular movement	Excretion of waste	

Food chemistry

- Food is composed of four broad classes of biomolecules that are broken down by the digestive system
    - Proteins
    - Fats
    - Polysaccharides
    - Nucleic Acids
- Biomolecules are chemically broken down into smaller components by enzymes
    - Proteins
        - Proteins are broken down by **proteases** into amino acids
        - This occurs in the stomach and small intestine
        - There are 20 amino acids. Humans make 11 of them on their own but need to acquire 9 through food
        - Proteins do the **'work'** in your body. They produce chemicals and facilitate chemical reactions in the cell
        - We are modelling protein digestion in this lab
    - Fats
        - Fats are broken down into fatty acids and monoglycerides by **lipases** in the small intestine
        - Fats are for **long term energy storage**
    - Polysaccharides
        - Polysaccharides (starch) and disaccharides (lactose) are broken down by **glycoside hydrolases** like amylase
        - Glycoside hyrdrolases turn long and complex sugars into disaccharides or monosaccharides like glucose and maltose which are then absorbed
        - Breakdown occurs in the small intestine
        - Carbohydrates are used as **short-term energy storage** or are converted into glycogen in the liver for future use
    - Nucleic acids
        - DNA (and RNA) **store genetic information** and are present in all cells
        - Nucleic acids start being broken down in the stomach with the acidic environment and enzymatically broken down by **nucleases** in the small intestine
        - The monomers of DNA can be taken back up into cells to synthesize new DNA during cell division

Digestion

- Digestion is the process the body goes through to turn food into nutrients.
- There are two methods of digestion that work together to break down food into its constituent components
    - Mechanical digestion starts right away when you chew your food, reducing larger pieces of food into smaller pieces. Peristalsis, the contraction and relaxation of the digestive muscles that move particles through your digestive tract.
    - Chemical digestion happens in concert with mechanical digestion and is

when enzymes chemically break down food.

- The Digestive Pathway
    - In your mouth, saliva contains amylases that start to chemically break down starch into monosaccharides.
    - Your stomach is very acidic and the enzyme pepsin thrives there, using the stomach acid to become more active and cleave the peptide bonds of proteins
    - Trypsin and chymotrypsin are active in the small intestine and are made in your pancreas
- Major proteases
    - Pepsin - activated by stomach acid, cleaves peptide bond after large amino acids
    - Trypsin - secreted by pancreas into small intestine, cleaves peptide bond after lysine and arginine
    - Chymotrypsin - secreted by pancreas into small intestine, cleaves peptide bond after large amino acids
- Papain
    - Digestive enzyme (protease) found in papaya fruit - pineapples have a similar protease (bromelain)
    - We are using papain as a digestive enzyme similar to trypsin
    - Papain has broad specificity - it cleaves after leucine and glycine but also hydrolyses esters and amides which allow it to rapidly breakdown complex molecules

Food Used in this Lab

- All foods have all four major biomolecules present inside them. However, some are more represented than others in a particular food
    - Bread - majority polysaccharides but structure is entirely from gluten, which is a protein. Bread is a great visual example of protease breakdown because the structure rapidly deteriorates
    - Spinach - lots of fiber, protein, polysaccharides
    - Egg - large protein content
    - Sweets - mostly simple sugars or polysaccharides
    - Banana – polysaccharides and fiber for structure

3rd Class:

Applications

Health and wellbeing

- Nutrition and healthy digestion are essential for maintaining bodily homeostasis and an overall sense of wellbeing.
- Deficiencies/diseases
    - Nutritional deficiencies such as anemia, vitamin B and Vitamin D deficiency, commonly occur due to restrictions in diet and can easily be treated via supplementation.
    - Some deficiencies may be a result of inherent causes such as abnormalities in blood cell function, enzymatic function or an inability to

absorb specific nutrients from foods.

- Chronic diseases such as Celiac Disease, Inflammatory Bowel Disease (IBD) or Crohn's Disease, if left untreated, lead to malnutrition, weight loss, exhaustion and depression.
- In Celiac Disease the immune system is triggered by gluten, protein found within some carbohydrates. The lining of the small intestine is degraded which leads to flattened and/or damaged microvilli (see images below and worksheet). Symptoms include malnourishment, diarrhea, cramps, weakened immune system and in children stunted growth. Effects can be reversed by adopting a strict gluten free diet.

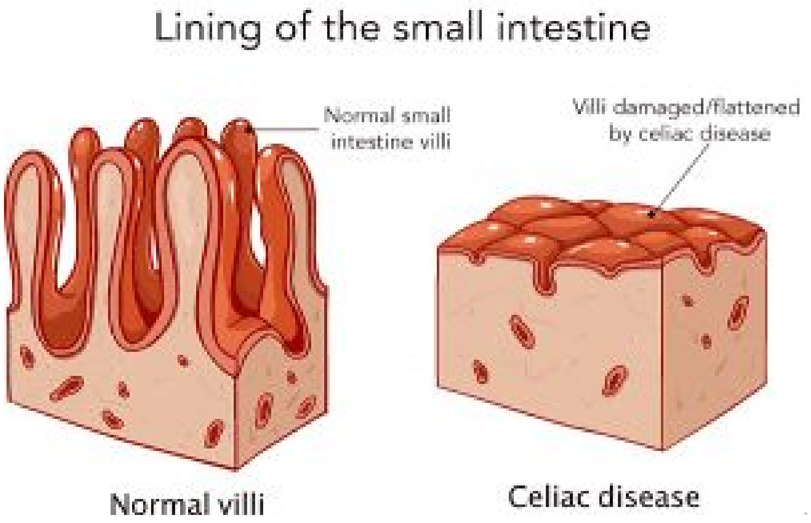

Intestinal microvilli in healthy and diseased conditions (beyondceliac.org)

- Gut microbiome
    - Digestive dysfunction can have a great impact on the mood and psychological well being. This area of research, termed the gut-brain-axis (see image below), has received a lot of interest in the scientific community in recent years.
    - Interestingly, 95% of the chemical serotonin (5HT), which is naturally made in our bodies, responsible for feelings of pleasure and wellbeing and used to treat depression, is actually found in our gut!

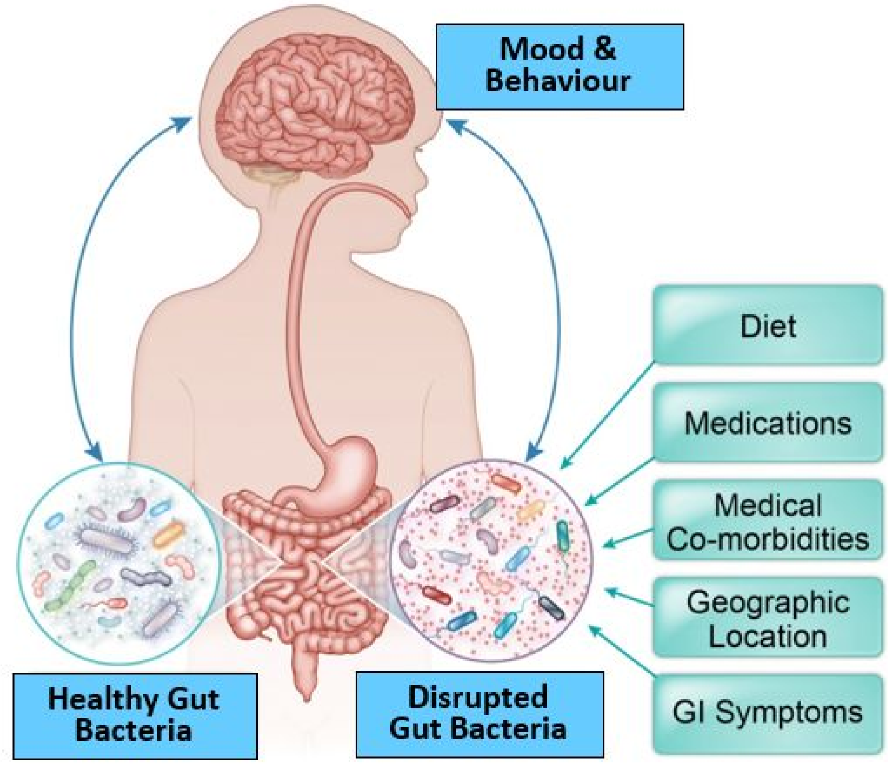

Gut-Brain-Axis connections (adapted from Saurman, V., Margolis, K.G. & Luna, R.A. Autism Spectrum Disorder as a Brain-Gut-Microbiome Axis Disorder. *Dig Dis Sci* 65, 818–828 (2020). DOI:10.1007/s10620-020-06133-5)

- Aside from the chemicals produced in our gut, many microorganisms thrive and 'feed' on carbohydrate/fibrous rich foods and release nutrients into our gut through an anaerobic process called fermentation.
- The microorganisms living in our gut are individual to each person, like a finger print, and collectively are called the gut microbiome.
- During fermentation these microorganisms release gas, which is why after eating a fibrous meal you may feel bloated!

- Fermentation in the food industry
    - Bacteria and yeast are also used outside of our bodies to produce fermented foods and beverages such as sourdough bread, dairy and alcohol.
    - The fermentation process has been used for thousands of years in many cultures to help purify and preserve food.
    - It is now known that fermented foods also improve the nutritional content of meals and feed the 'good' bacteria in our guts.

Preparation:

1st Class:

- Divide students into groups (2 - 4 students per group).

- Set out materials for each group. Each group will need:
    - >6g of each food type (bread, sweets, spinach, banana, egg [optional - hard-boiled, white-only]),
    - 100mL of papain solution (1g of papain powder into 350mL of water - can be prepared in bulk for class or individually by students),
    - 100 mL of water,
    - 12 glass vials with caps.

Learning Objectives

AQA GCSE:

- Students should be able to relate knowledge of enzymes to Metabolism.
- Students should be able to describe the nature of enzyme molecules.
- Students should be able to recall the sites of production and the action of amylase, proteases and lipases.
- Students should be able to understand simple word equations but no chemical symbol equations are required. Digestive enzymes convert food into small soluble molecules that can be absorbed into the bloodstream. Carbohydrase break down carbohydrates to simple sugars. Amylase is a carbohydrase which breaks down starch. Proteases break down proteins to amino acids. Lipases break down lipids (fats) to glycerol and fatty acids.
- The products of digestion are used to build new carbohydrates, lipids and proteins. Some glucose is used in respiration. Bile is made in the liver and stored in the gallbladder. It is alkaline to neutralize hydrochloric acid from the stomach. It also emulsifies fat to form small droplets which increases the surface area. The alkaline conditions and large surface area increase the rate of fat breakdown by lipase.
- (Higher tier GSCE only) The digestion of proteins from the diet results in excess amino acids which need to be excreted safely. In the liver these amino acids are deaminated to form ammonia. Ammonia is toxic and so it is immediately converted to urea for safe excretion.

NGSS:

- HS-LS1-2: Develop and use a model to illustrate the hierarchical organization of interacting systems that provide specific functions within multicellular organisms.
- HS-LS1-6: Construct and revise an explanation based on evidence for how carbon, hydrogen, and oxygen from sugar molecules may combine with other elements to form amino acids and/or other large carbon-based molecules.
- HS-LS1-7: Use a model to illustrate that cellular respiration is a chemical process whereby the bonds of food molecules and oxygen molecules are broken and the bonds in new compounds are formed resulting in a net transfer of energy.

Resources:

Digestion Lab Demonstration (University of Cambridge) *March 2021*
https://www.youtube.com/watch?v=H31oWygVsho

Your Digestive System & How it Works (US NIH) *December 2017*

https://www.niddk.nih.gov/health-information/digestive-diseases/digestive-system-how-it-works

Digestive System: Function, Organs & Anatomy (Cleveland Clinic) *August, 2018*
https://my.clevelandclinic.org/health/articles/7041-the-structure-and-function-of-the-digestive-system

Anatomy, Abdomen and Pelvis, Small Intestine (US NIH) *August 2021*
https://www.ncbi.nlm.nih.gov/books/NBK459366/

Probing the Histories of Human Digestion (Science History Institute) *August 2018*
https://www.sciencehistory.org/distillations/probing-the-mysteries-of-human-digestion

Digestive System (BBC) *Accessed October 2021*
https://www.bbc.co.uk/bitesize/guides/zwqycdm/revision/4

Funding:

Biochemical Society of the UK

Acknowledgements:

Jesus College, University of Cambridge
Department of Chemical Engineering and Biotechnology, University of Cambridge
Cancer Research UK, Cambridge Institute
