# Supplementary material for: Digesting Digestion: An Educational Laboratory to Teach Students about Enzymes and the Gastrointestinal Tract
Source: J Chem Educ. 2023 Jan 19;100(2):907–13. doi: 10.1021/acs.jchemed.2c00992 (PMC9933529; doi:10.1021/acs.jchemed.2c00992)
Supplement: Supplementary file 9 — ed2c00992_si_009.pdf [file ed2c00992_si_009.pdf]

Supporting Information

Question & Answers Sheet

Digesting digestion: An educational laboratory to teach students about enzymes and the gastrointestinal tract

Stephanie Mack[1], Sarah L. Barron[2], Alexander J. Boys[2]*

1. Cancer Research UK Cambridge Institute, University of Cambridge, Robinson Way, Cambridge CB2 0RE, United Kingdom

2. Department of Chemical Engineering and Biotechnology, University of Cambridge, Philippa Fawcett Drive, Cambridge, CB3 0AS, United Kingdom

* Corresponding Author (ab2661@cam.ac.uk)

Question & Answers Sheet – Live Q&A (For Teacher Only)

1. Why did we use water and papain vials for this experiment?
   The water was used as a control so we can observe what the papain does in context.

2. Digestion occurs through two major means, mechanical and chemical. What is the purpose of each of these means?

   Mechanical
   Mechanical digestion physically breaks food down into smaller pieces.

   Chemical
   Chemical digestion reduces biomolecules into its basic units.

3. Which types of biomolecule does your stomach primarily digest?
   Your stomach digests proteins.

4. Name an enzyme found in plants.
   Papain is an enzyme found in plants.

   Why would a plant excrete an enzyme like this?
   Defense against pests, digests flies and other herbivorous insects.

5. What would happen if we changed the pH of the water in this laboratory?
   The pH change may change the action of the enzyme, rendering it more or less effective. If the pH change is acidic, the acidic environment could further break down food. If the pH change was basic, the basic environment could also break up food but less efficiently.

6. Can you think of another use for enzymes to help with more global problems (apart from breaking down foods, etc.)?
   Mention environmental waste – digestion of plastics, sanitation or water purification. Artificial enzymes could else help treat diseases by acting on the infectious agent.
